# The fecal microbiota of healthy dogs fed extruded and raw meat-based diets

**DOI:** 10.3389/fmicb.2026.1910251

**Published:** 2026-09-25

**Authors:** Doina Solís, Magaly Toro, Paola Navarrete, Daniel A. Medina, Angélica Reyes-Jara

**Affiliations:** 1 Laboratorio de Microbiología y Probióticos, Instituto de Nutrición y Tecnología de los Alimentos (INTA), Universidad de Chile, Avenida El Líbano, Macul, Santiago, Chile; 2 Millenium Institute Center for Genome Regulation (CGR), Santiago, Chile; 3 Joint Institute for Food Safety and Applied Nutrition (JIFSAN), University of Maryland, College Park, MD, United States; 4 Escuela de Química y Farmacia, Facultad de Ciencias, Universidad San Sebastián, Puerto Montt, Chile; 5 Laboratorio Institucional, Universidad San Sebastián, Puerto Montt, Chile

**Keywords:** dog microbiota, one health, pathogen carriage, pet food, pet nutrition, zoonoses

## Abstract

Conventional dog diets, such as extruded diets, remain common. The popularity of unconventional options, particularly raw diets, has increased over time and may differentially influence the gut microbiota. Raw diets inherently harbor higher bacterial loads, including potentially pathogenic microorganisms, raising concerns from a One Health perspective due to the close interaction among pets, humans, and the environment. The study aims were to characterize the nutritional profiles of extruded and raw diets, compare the fecal microbial composition of dogs fed extruded diets with that of dogs fed RMBDs, and evaluate microbiota differences in pathogen-carrying dogs. Extruded (*n* = 10) and raw (*n* = 6) diets were analyzed. Fecal samples from extruded-fed (*n* = 14) and raw-fed dogs (*n* = 10), including samples from raw-fed dogs previously identified as pathogen carriers (6/10), were analyzed. The V3–V4 region of the 16S rRNA gene was sequenced, and diversity metrics were obtained to evaluate intra- and inter-sample variations. RMBDs showed higher protein and fat contents and lower fiber and non-fiber carbohydrate levels compared to extruded diets. Discrepancies between declared and analyzed nutritional composition were found in 100% of raw diets and 10% of extruded diets. Alpha diversity showed a mixed pattern between dietary groups, with significant differences in Chao1 and Simpson indices but not across all diversity metrics. Raw-fed dogs showed higher microbial diversity and increased relative abundance of specific taxa. Taxa differences between raw- and extruded-fed dogs suggest an association with diet composition. Raw-fed dogs identified as pathogen carriers showed significantly lower microbial diversity than non-carriers. These findings highlight the variability and potential nutritional imbalances of raw diets, confirm diet as a key modulator of canine gut microbiota, and suggest that greater bacterial diversity does not necessarily reflect healthier microbiota.

## 1 Introduction

The canine gut microbiota, comprising bacteria, archaea, viruses, and eukaryotic microorganisms, is shaped by factors such as environment, veterinary care, immune status, antimicrobial use, and nutrition (Liu et al., 2021; Suchodolski, 2021). These influences modulate key host functions, including metabolism, immune regulation, and anti-inflammatory responses (Huang et al., 2020). Alterations in the microbiota are linked not only to intestinal dysfunction but also to systemic diseases in dogs and cats, such as chronic kidney disease, cardiac conditions, obesity, endocrine and neurologic disorders (Suchodolski, 2021; Pilla and Suchodolski, 2020). Diet is directly related to human and animal gut microbiota since nutrients might modify microorganisms and metabolites present in the gastrointestinal tract (Huang et al., 2020; Kim et al., 2017). Canine dietary habits have suffered modifications through time, and nowadays owners have different feeding options to choose from, including, among others, extruded and raw diets. Each diet is made with different ingredients and through different processes that not only contribute to the nutritional composition and palatability of pet foods, but also to petfood safety (Crane et al., 2010; Dodd et al., 2020; Solís et al., 2022; Buff et al., 2014; Geary et al., 2024).

The composition of the gut microbiota has been predominantly studied in human and murine models, whereas research in small animals such as dogs remains limited. Nevertheless, available studies indicate that the canine gut microbiota exhibits the greatest taxonomic and functional overlap with the human intestinal microbiota (Hernandez et al., 2022) A more detailed understanding of the composition of the canine gut microbiota may improve our knowledge of how the diet influences their microbiota.

The predominant phyla in the gut microbiota of dogs are Bacillota, Bacteroidota, and Fusobacteriota (Hernandez et al., 2022; Hiney et al., 2024; Suchodolski, 2021; Kim et al., 2025). Nonetheless, the composition of the gut microbiota may suffer modifications due to dietary habits that might contribute to changes in the bacterial abundance and diversity (Hernandez et al., 2022). Some studies have reported that dogs consuming natural diets, generally understood as diets with a lower degree of processing and fewer additives, although not standardized in veterinary medicine (Buff et al., 2014), have a more diverse and abundant gut microbiota than dogs fed commercial extruded diets (Huang et al., 2020; Kim et al., 2017; Hiney et al., 2024).

In dogs, it has also been observed that protein-rich diets, such as raw meat-based diets (RMBD), are related to increases in the abundance of *Clostridiaceae* bacterial family, for instance *Clostridium perfringens* and *Clostridioides difficile*, as well as increases in the abundance of microorganisms from the Fusobacteriota and Bacteroidetota phyla (Pilla and Suchodolski, 2020; Martínez-López et al., 2021).

Moreover, the integrity of the gastrointestinal tract, particularly the mucosal barrier, and consequently the composition of the gut microbiota, may be disrupted by the colonization with zoonotic microorganisms, such as enteropathogenic *Escherichia coli, Salmonella, Listeria monocytogenes*, among others (Viswanathan et al., 2009; Dróżdż et al., 2021; Drolia et al., 2018). Nevertheless, given that enteropathogens have been recovered from the gastrointestinal tract of asymptomatic animals, further research is needed to understand the impact these microorganisms may have on the canine gut microbiota composition (Solís et al., 2022; Schmitz and Suchodolski, 2016; Sykes, 2023).

Therefore, given the strong influence of diet on the gut microbiota and the different feeding formats currently available for pets, this study aims to characterize the nutritional composition of the diets, including the comparison between label declarations and analytical values, to investigate the effect of extruded and raw meat-based diets on the composition of the gut microbiota of clinically healthy dogs, and to evaluate the association between pathogen carriage and microbiota modulation.

## 2 Materials and methods

### 2.1 Fecal samples

Fecal samples from a total of 24 dogs (extruded-fed = 14; raw-fed = 10) from a previously reported study (Solís et al., 2022) were used, and two extruded-fed dogs were included as new study participants. Of all the participants, six were pathogen carriers (*Salmonella* = 5; *L. monocytogenes* = 1), which belonged to the group of pets fed a raw diet. Prior to the sampling, the dog’s health status, including a general physical examination and the assessment of the pets’ body condition and muscle score, was assessed by veterinarians. Only healthy dogs with a healthy body condition and without acute gastrointestinal symptoms, such as vomiting or diarrhea, were enrolled in the study. Also, diet-specific factors, such as the type of diet consumed, were gathered. Besides the health status, exclusion criteria included undergoing medical therapy, such as antibiotics and/or corticosteroids, the use of probiotics for at least 6 weeks before the sample collection, and the use of mixed diets (combined raw and extruded diet). The fecal samples were retrieved from the sample bank of the Microbiology and Probiotics Laboratory (INTA, University of Chile) where they were kept at −80 °C until the DNA extraction.

### 2.2 Diet composition

To assess the nutritional composition of the RMBDs and extruded diets consumed by the dogs enrolled in the study, a proximate nutritional analysis was performed to six of the raw meat-based diets consumed by the participants and 10 extruded canine diets, including the analysis of: (1) moisture, (2) ash content, (3) crude protein, (4) fat, (5) fiber, and (6) the available carbohydrates were obtained by difference (Montegiove et al., 2022; Dobenecker and Kienzle, 1998). The results obtained were compared with the information stated on each product’s label. Deviations were reported for those results that exceeded the maximum tolerance permitted by Regulation (EC) No. 767/2009 of the European Parliament and of the Council, as of December 26, 2018 (European Parliament and Council of the European Union, 2018).

### 2.3 Bacterial DNA extraction and 16S rRNA gene amplicon sequencing

Fecal samples previously used for pathogen identification were afterwards used for microbiota characterization by 16S rRNA gene sequencing. Sequencing procedures were initiated immediately following pathogen analysis, with samples kept at −80 °C until DNA extraction (Solís et al., 2022). Thus, pathogen detection and microbiota characterization were performed on the same fecal specimens, ensuring that both analyses represented the same biological sampling time point. Microbial genomic DNA was extracted from fecal samples with DNeasy^®^ PowerSoil^®^ (Qiagen, Germany). Briefly, 0.25 g of fecal samples were added to PowerBead Tubes and mixed. The protocol was followed according to the manufacturer’s instructions, and the total DNA was eluted in sterile elution buffer. Each DNA sample was analyzed with the NanoDrop Microvolume Spectrophotometer (Thermo Fisher, United States) for the assessment of adequate A260/A280 and A260/A230 purity ratios and the concentration of nucleic acids. The extracted DNA was stored at −20 °C until further analysis was conducted. The amplification and sequencing of the V3–V4 region of the 16S rRNA gene was performed by an external provider (Novogene, United States) using the primers 341F (5’-CCTAYGGGRBGCASCAG-3′) and 806R (5’-GGACTACNNGGGTATCTAAT-3′) (Luecke et al., 2024) for amplification carried out on Illumina platforms.

### 2.4 Bioinformatics and statistical analysis

Nutrient composition variables (ash, crude protein, crude fat, crude fiber, and non-fiber carbohydrates) were analyzed on a dry matter (DM) basis, while metabolizable energy (ME) was expressed as-fed (AF). To assess overall differences in nutrient composition between raw and extruded diets, a multivariate analysis of variance (MANOVA) was conducted using diet type as the fixed factor and ash, crude protein, crude fat, and crude fiber as dependent variables. Non-fiber carbohydrates (NFC) were not included in the multivariate model since they were calculated by difference from the other proximate components, thereby avoiding mathematical redundancy among dependent variables. Pillai’s trace was used as the multivariate test statistic because of its relative robustness to moderate departures from multivariate assumptions (Rencher and Christensen, 2012).

Individual nutrient concentrations, including ash, crude protein, crude fat, crude fiber, and NFC, were compared between diet types using Welch’s two-sample *t*-tests, which do not assume equal variances between groups (West, 2021). To account for multiple nutrient-level comparisons, *p*-values were adjusted using the Benjamini–Hochberg false discovery rate (FDR) procedure (Benjamini and Hochberg, 1995). A leave-one-out case-deletion sensitivity analysis was performed to assess the robustness of the overall diet-type effect to the exclusion of individual RMBDs. The MANOVA was sequentially repeated after excluding each of the six RMBDs individually while retaining all 10 extruded diets. Pillai’s trace, *F*-statistics, and *p*-values were recorded for each iteration to assess the stability of the overall diet-type effect and determine whether the observed multivariate difference was disproportionately influenced by any single RMBD (Rencher and Christensen, 2012; Tang and Fung, 1997).

Principal component analysis (PCA) was performed using centered and standardized ash, crude protein, crude fat, and crude fiber concentrations to visualize multivariate patterns in nutrient composition between diet types and identify the nutrients contributing most strongly to the principal components. Statistical significance was set at *p* < 0.05. Statistical analyses were performed using RStudio [version 2026.01.0] (Rencher and Christensen, 2012).

Multiple alpha diversity indices were calculated as each metric captures distinct aspects of community diversity and structure (Chao and Chiu, 2016; Thukral, 2017; Cassol et al., 2025). Alpha diversity indices were calculated for dogs fed raw diets and subsequently stratified according to pathogen carriage (pathogen carriers vs. non-pathogen carriers). Alpha diversity was assessed using the Observed species, Chao1, ACE, Shannon, Simpson, and Fisher indices (Li et al., 2022). For normally distributed indices with homogeneous variances, differences between dietary groups were evaluated using an independent-samples Student’s *t*-test (Borman et al., 2021; Shao et al., 2020). When homogeneity of variance was not met, the degrees of freedom were adjusted using the Welch-Satterthwaite approximation, whereas the Wilcoxon rank-sum test was used when the normality assumption was not met (Ahad and Yahaya, 2014). Differences in alpha diversity indices between pathogen carriers and non-pathogen carriers within the raw-fed group were evaluated using the Mann–Whitney *U* test. Results were considered statistically significant at *p* < 0.05.

Core taxa were defined as those present in at least 80% of samples within each dietary group at a relative abundance detection threshold of 0.0001 (0.01%).

Beta diversity was evaluated by weighted and unweighted UniFrac distance matrices and presented with a principal coordinate analysis (PCoA) plot (You and Kim, 2021). PERMANOVA was used to assess differences in microbiota composition.

Because puppies were present exclusively in the extruded-fed group, a sensitivity analysis was conducted to assess the potential influence of age on the observed microbiota differences. The three puppies were excluded, and alpha and beta diversity analyses were repeated using only adult and senior dogs (raw-fed, *n* = 10; extruded-fed, *n* = 11), following the same statistical procedures described above. For beta diversity analyses, homogeneity of multivariate dispersion was assessed using PERMDISP.

Bioinformatic analysis was performed using the Phyloseq object obtained from DADA2 analysis (Callahan et al., 2017) in R version 4.1.0, and the original taxon-level screening was performed with the run_lefse function implemented in microbiomeMarker version 1.3.3 (Cao et al., 2022). Relative-abundance data were analyzed separately at the phylum, family, and genus levels using the original LEfSe criteria (Kruskal–Wallis *p* < 0.05, Wilcoxon *p* < 0.05, multigrp_strat = TRUE, and LDA score ≥ 2). To assess the robustness of these taxon-level findings to multiple testing, Kruskal-Wallis *p*-values were subsequently recalculated for the complete set of taxa evaluated at each rank (35 phyla, 240 families, and 477 genera), and Benjamini–Hochberg false-discovery-rate correction was applied independently within each taxonomic rank. The archived original LEfSe LDA scores and group assignments were retained, and taxa meeting the original LEfSe criteria but not BH *q* < 0.05 were interpreted as nominally discriminative exploratory signals rather than FDR-supported differential taxa. Original and recalculated *p*-values, BH-adjusted *q*-values, LDA scores, group assignments, and within-group abundance summaries are provided in Supplementary Table S1. LEfSe effect-size plots were generated from the original archived LEfSe results using ggplot2 (Wickham, 2016).

To evaluate whether differences in sequencing depth could confound the observed alpha-diversity patterns, final library size was quantified for each sample as the number of non-chimeric reads retained after DADA2 denoising, paired-end merging, and chimera removal. Differences in sequencing depth between dietary groups were assessed using Welch’s *t*-test on log10-transformed read counts. The relationship between sequencing depth and richness was further evaluated using Spearman’s rank correlation for Observed richness and Chao1. In addition, linear models including diet and log10-transformed sequencing depth as predictors were fitted independently for Observed richness and Chao1 (richness ~ diet + log10 sequencing depth). These analyses were performed to determine whether sequencing effort contributed to the observed differences in richness between dietary groups. Per-sample sequencing depths and richness estimates are provided in Supplementary Table S2. Primary diversity analyses were conducted using the non-rarefied dataset, following recommendations against routine rarefaction and after explicitly evaluating sequencing depth as a potential confounding factor (McMurdie and Holmes, 2014).

## 3 Results

### 3.1 Demographics of dogs enrolled in the study

The demographics of the 24 study participants, including sex, life stage and the represented breeds are shown in Table 1.

**Table 1:** Demographics of dogs enrolled in the study (*n* = 24).

Variable	Number of RMBD-fed dogs (*n* = 10), *n* (%)	Number of extruded-fed dogs (*n* = 14), *n* (%)	Total (*n* = 24), *n* (%)
Sex			
Female	6 (60)	9 (64.3)	15 (62.5)
Male	4 (40)	5 (35.7)	9 (37.5)
Life stage			
Puppy^1^	0 (0)	3 (21.4)	3 (12.5)
Adult^2^	9 (90)	10 (71.4)	19 (79.2)
Senior^3^	1 (10)	1 (7.14)	2 (8.3)
Breeds			
Mixed-breed	5 (50)	5 (35.7)	10 (41.7)
Pug	0 (0)	2 (14.3)	2 (8.3)
Dachshund	1 (10)	0 (0)	1 (4.2)
Akita	1 (10)	0 (0)	1 (4.2)
Bernese Mountain Dog	0 (0)	1 (7.1)	1 (4.2)
Border Collie	0 (0)	1 (7.1)	1 (4.2)
Miniature Schnauzer	0 (0)	1 (7.1)	1 (4.2)
Chow Chow	1 (10)	0 (0)	1 (4.2)
Airedale Terrier	0 (0)	1 (4.2)	1 (4.2)
Golden Retriever	0 (0)	1 (7.1)	1 (4.2)
Labrador Retriever	0 (0)	1 (7.1)	1 (4.2)
Shih Tzu	0 (0)	1 (7.1)	1 (4.2)
Yorkshire Terrier	1 (10)	0 (0)	1 (4.2)
West Highland White Terrier	1 (10)	0 (0)	1 (4.2)

^1^Puppy category: dogs under 1 year of age.

^2^Adult category: dogs between 1 and 7 years.

^3^Senior category: dogs over 7 years.

### 3.2 Diet composition

The comparison of the nutritional content between the analyzed canine extruded diets (n = 10) and canine raw meat-based diets (*n* = 6) is shown in Table 2. RMBDs differed significantly from extruded diets in their overall nutrient composition (MANOVA, Pillai’s trace = 0.977, *F*(4,11) = 118.86, *p* < 0.001) (Supplementary Figure S1), exhibiting higher protein and fat contents and lower levels of non-fiber carbohydrates (NFC) and fiber. Nutrient-specific tests showed that RMBDs had significantly higher crude protein (FDR-adjusted *p* = 6.07 × 10^−5^) and crude fat (*p* = 1.56 × 10^−4^), and significantly lower crude fiber (*p* = 7.60 × 10^−6^) and non-fiber carbohydrate concentrations (*p* = 9.10 × 10^−7^) than extruded diets. Ash content did not differ between diet types (*p* = 0.875). These differences were associated with significantly lower metabolizable energy in RMBDs when expressed on an as-fed basis (AF) compared to extruded diets (*p* < 0.001). The description of the ingredients used in the raw diets can be found in Supplementary Table S3.

**Table 2:** Comparison of the nutritional content of pet foods on a dry basis (100 g/dry matter).

Calculated nutrients (mean)	Analyzed diets	
	Raw diets (*n* = 6)	Extruded diets (*n* = 10)
Ash (g)	8.1 ± 2.3	8.3 ± 1.2
Crude protein (g)^1^	51.2 ± 5.9	26.8 ± 3.3
Total fat (g)^1^	28.9 ± 4.2	12.7 ± 1.6
Crude fiber (g)^1^	1.1 ± 0.4	3.4 ± 0.6
Non-fiber carbohydrates (g)^1^	10.2 ± 5.2	48.9 ± 3.8
Energy (kcal/AF)^2^	143.7 ± 23.3	364.9 ± 24.3

Values are presented as mean ± SD. Nutrient concentrations are expressed on a dry matter (DM) basis, whereas metabolizable energy (ME) is expressed on an as-fed (AF) basis.

MANOVA (Pillai’s trace = 0.977, *F*(4,11) = 118.86, *p* < 0.001).

^1^FDR-adjusted *p* < 0.001.

^2^Welch’s *t*-test (11.07) = −18.11, *p* < 0.001.

Furthermore, the nutritional analysis of raw diets revealed deviations with respect to the tolerances established in Regulation (EC) No 767/2009 (v. 26.12.2018) (European Parliament and Council of the European Union, 2018), which sets labeling requirements and permissible analytical tolerances for pet foods marketed in the European Union. The discrepancies were particularly observed for moisture, crude protein, crude fat, and metabolizable energy (ME) in all the RMBDs analyzed (6/6). In contrast, extruded diets showed a high level of agreement with label declarations, with only a single deviation observed in the crude fat content of one sample (1/10) (Supplementary Table S4).

### 3.3 Microbiota composition of raw and extruded-fed dogs

A total of 4,142,464 raw reads were obtained from the 24 canine fecal samples. After quality filtering, 3,894,351 reads were retained (raw-fed dogs = 1,569,029; extruded-fed dogs = 2,325,322). Following DADA2 denoising, paired-end merging, and chimera removal, 2,310,956 non-chimeric reads were retained for downstream analyses (raw-fed dogs = 987,259; extruded-fed dogs = 1,323,697). Final sequencing depth ranged from 26,479 to 128,888 reads per sample. Mean sequencing depth was 98,726 reads in raw-fed dogs and 94,550 reads in extruded-fed dogs (Supplementary Table S5), and did not differ significantly between dietary groups (Welch’s *t*-test on log10-transformed read counts, *p* = 0.959). All samples showed adequate sequencing coverage based on the rarefaction curves (Supplementary Figure S2). Sequencing depth was not associated with Observed richness (Spearman’s *ρ* = 0.005, *p* = 0.982) or Chao1 richness (*ρ* = 0.035, *p* = 0.872). Consistently, in linear models accounting for both diet and sequencing depth, log10-transformed read depth was not significantly associated with either Observed richness (*p* = 0.498) or Chao1 (*p* = 0.484), whereas diet remained significantly associated with both Observed richness (*p* = 0.033) and Chao1 (*p* = 0.028). These results indicate that differences in sequencing effort did not account for the observed richness patterns between dietary groups. Therefore, downstream analyses were performed using the non-rarefied dataset to avoid unnecessary loss of sequence information (McMurdie and Holmes, 2014). A total of 35 phyla, 79 classes, 169 orders, 240 families, and 477 genera were identified.

### 3.4 Alpha diversity

Alpha diversity differed between dietary groups in a metric-dependent manner. Chao1 indicated significantly higher estimated richness in raw-fed dogs (*p* = 0.04; Welch’s *t*-test), whereas the Simpson index also differed significantly between groups (*p* = 0.02; Wilcoxon test) but showed a different directional pattern (Figure 1). Observed richness, Shannon, Fisher, and ACE did not differ significantly between dietary groups (*p* > 0.05). To assess the potential influence of age on the observed microbiota differences, a sensitivity analysis was performed after excluding the three puppies, which were present exclusively in the extruded-fed group. The analysis therefore included 10 raw-fed and 11 extruded-fed adult or senior dogs. The overall alpha diversity pattern was maintained after puppy exclusion. Chao1 (*p* = 0.046), ACE (*p* = 0.046), and Simpson (*p* = 0.009) differed significantly between dietary groups, whereas Observed species (*p* = 0.052), Shannon (*p* = 0.281), and Fisher (*p* = 0.051) did not (Supplementary Table S6). These results indicate that the alpha diversity differences observed between dietary groups were not primarily driven by the inclusion of puppies in the extruded-fed group.

**Figure 1:**
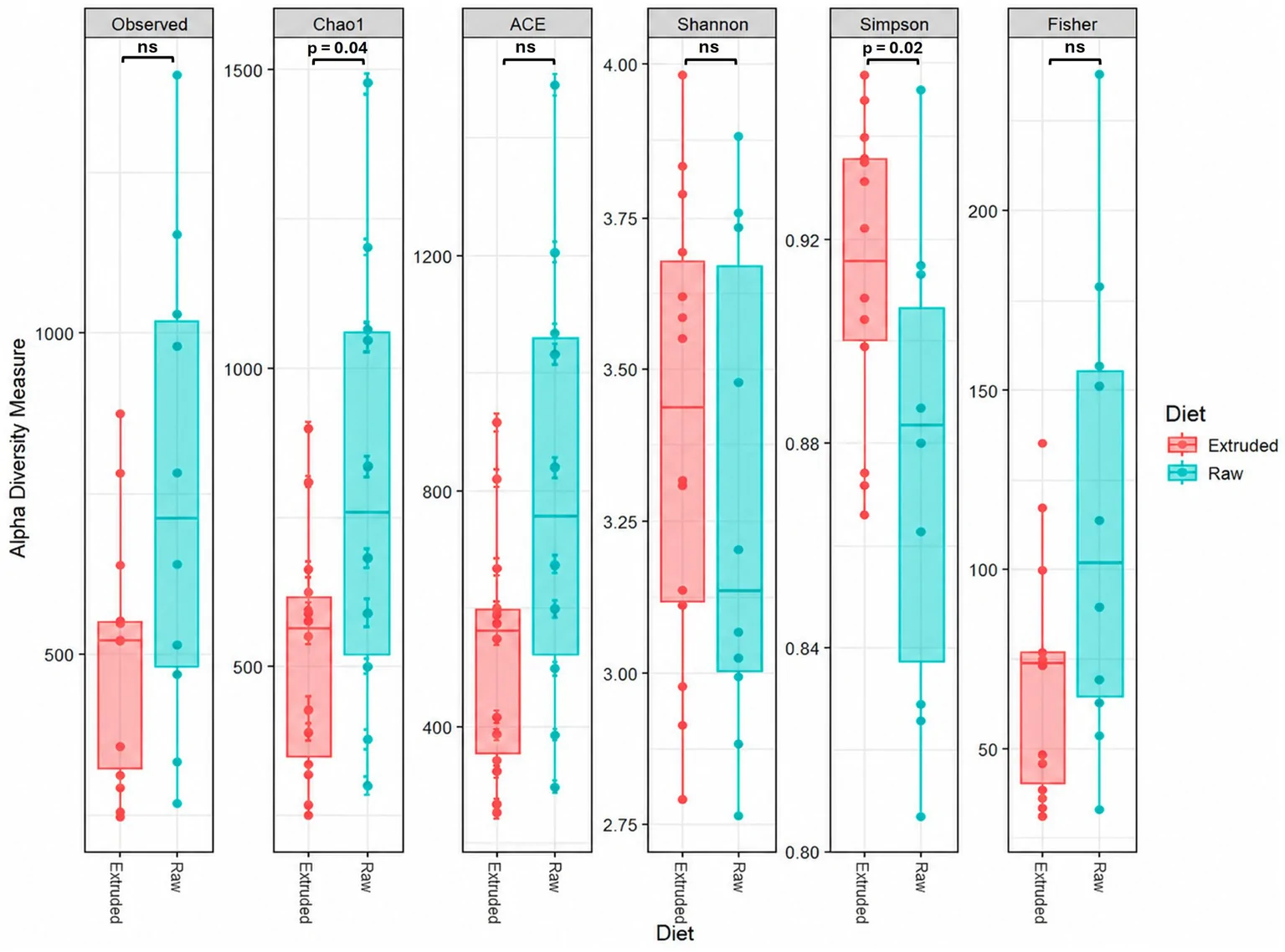
Comparison between alpha diversity indices for fecal samples obtained from extruded-fed dogs (*n* = 14) and raw (*n* = 10). Chao1 (*p* = 0.04; Welch’s *t*-test) and Simpson (*p* = 0.02; Wilcoxon test) revealed significant differences between groups. ns, not significant.

### 3.5 Beta diversity

Differences in microbial community composition between the extruded-fed and the raw group were visualized using principal coordinate analysis (PCoA) based on weighted UniFrac distances (*p* = 0.003; Figure 2). Beta diversity analysis based on unweighted UniFrac distances did not reveal statistically significant differences between groups (*p* = 0.07). Sensitivity analyses excluding the three puppies from the extruded-fed group returned a similar beta diversity pattern. Weighted UniFrac remained significantly associated with diet type (PERMANOVA: *R*^2^ = 0.168, *F* = 3.838, *p* = 0.006), whereas unweighted UniFrac remained non-significant (*R*^2^ = 0.072, *F* = 1.481, *p* = 0.094). No differences in multivariate dispersion were detected for either weighted (PERMDISP, *p* = 0.635) or unweighted UniFrac (*p* = 0.801) (Supplementary Table S7 and Supplementary Figure S3).

**Figure 2:**
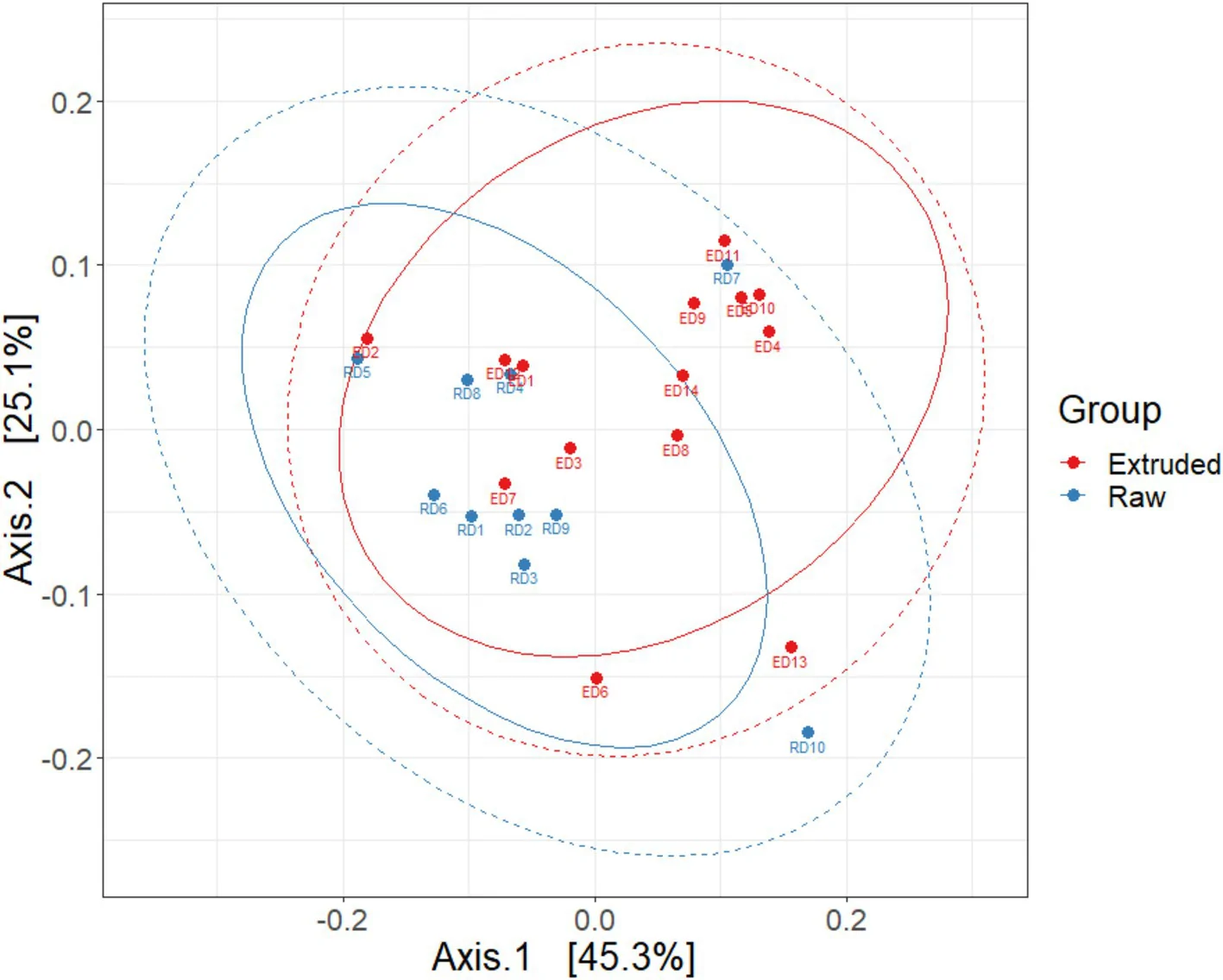
PCoA plot of beta diversity values based on weighted UniFrac distances. Comparison of the fecal microbiota from extruded-fed and raw-fed dogs. Dots represent the samples, and different colors represent different groups. The closer the distance between samples, the higher the similarity. Ellipses show confidence intervals (CI) of 95% for each sample type (*p* = 0.003; PERMANOVA analysis).

### 3.6 Taxonomic fecal microbiota composition

A shared core microbiota, which refers to a stable subset of microorganisms consistently present across individuals (Zhao, 2025), was identified in both dietary groups. This core community was primarily composed of members of the family Bacteroidaceae (*Bacteroides*), Lachnospiraceae (*Blautia*), Fusobacteriaceae (*Fusobacterium*), and Lachnospiraceae (*Lachnoclostridium*) (Figure 3).

**Figure 3:**
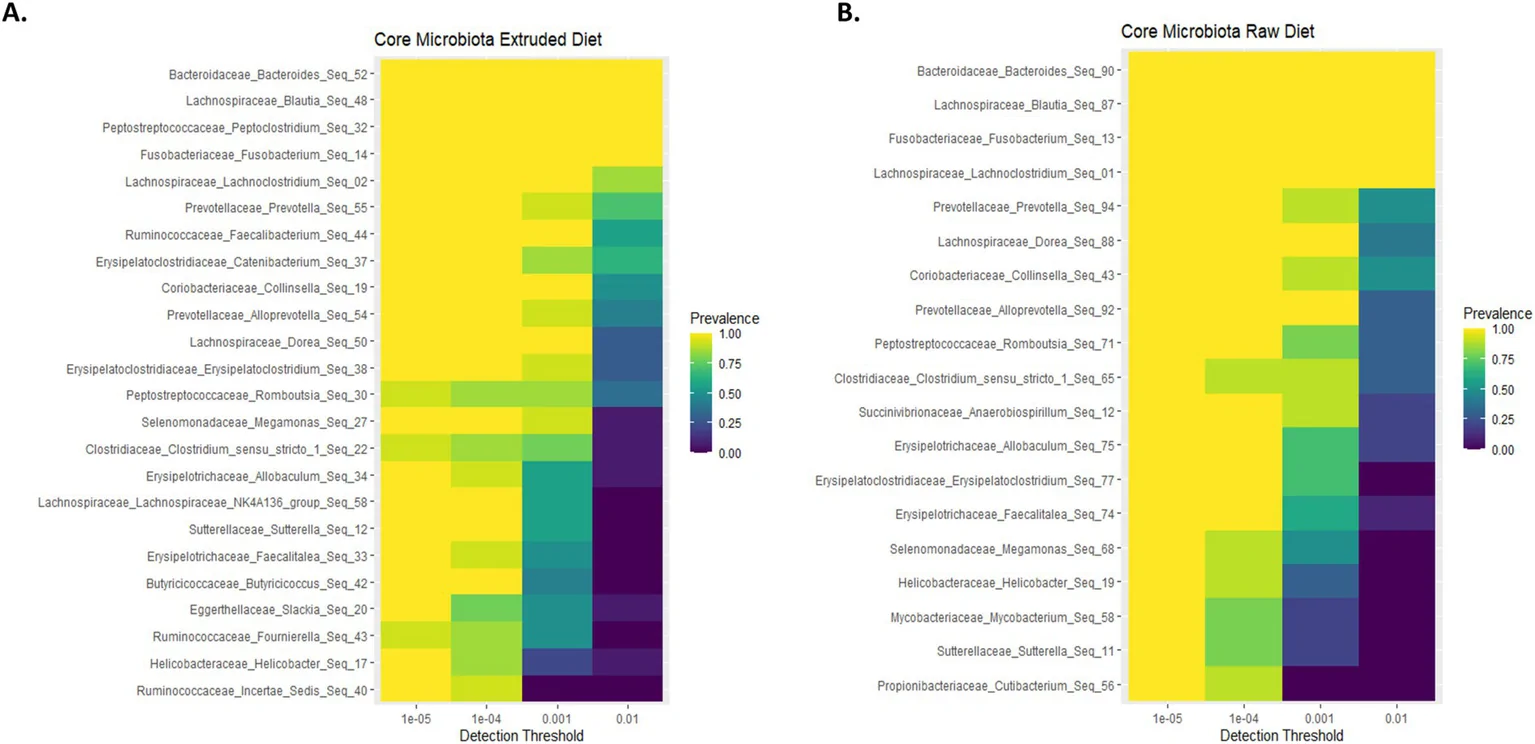
Core microbiota identified in the fecal samples obtained from extruded **(A)** and raw-fed dogs **(B)**. Each row represents a bacterial taxon, while the x-axis shows increasing detection thresholds. Color intensity (yellow) indicates a higher proportion of taxa across all samples (McMurdie and Holmes, 2013).

Regarding taxonomic abundance, at the phylum level, Bacillota, Fusobacteriota, and Bacteroidota dominated the gut microbiota of extruded and raw -fed dogs, accounting for over 90% of total relative abundance across all samples (Figure 4). Dogs fed raw diets showed a higher relative abundance of Fusobacteriota (39.8% ± 0.2) whereas Bacillota predominated in dogs fed extruded diets (43.4% ± 1.4) (Supplementary Table S5 and Figure 4).

**Figure 4:**
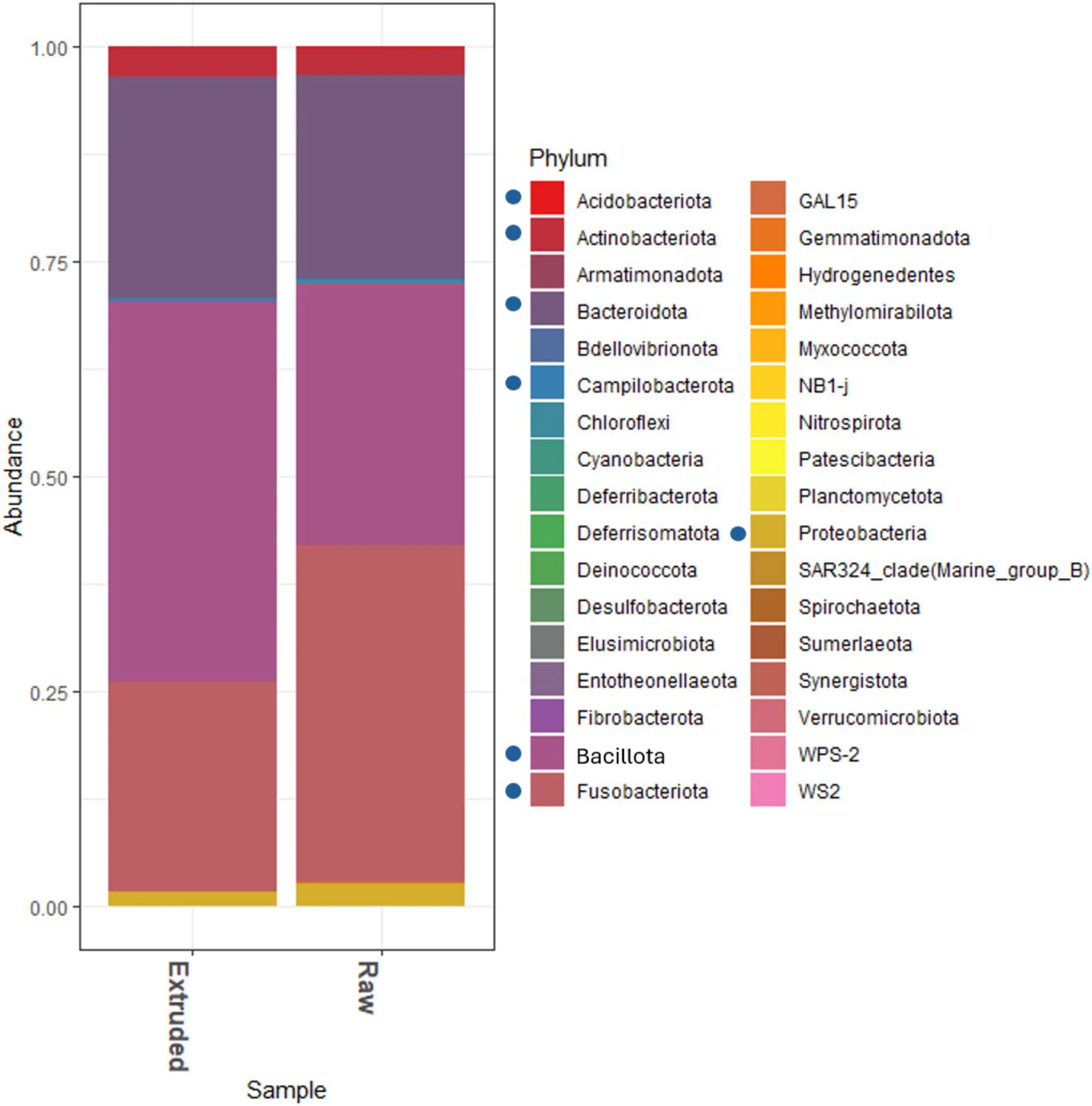
Identified phyla. Overview of the top bacterial phyla detected in the extruded and raw-fed groups. Blue circles indicate phyla shown in the figure.

Using the original nominal LEfSe criteria (Kruskal–Wallis *p* < 0.05, Wilcoxon *p* < 0.05, and LDA score ≥ 2), Bacillota was nominally discriminative for extruded-fed dogs, whereas Acidobacteriota, Cyanobacteriota, Chloroflexota, Myxococcota, and Verrucomicrobiota were nominally discriminative for raw-fed dogs (Figure 5 and Supplementary Table S1). After Benjamini–Hochberg correction across all 35 phylum-level tests, none of these signals remained significant (all *q* ≥ 0.05; minimum *q* = 0.066).

**Figure 5:**
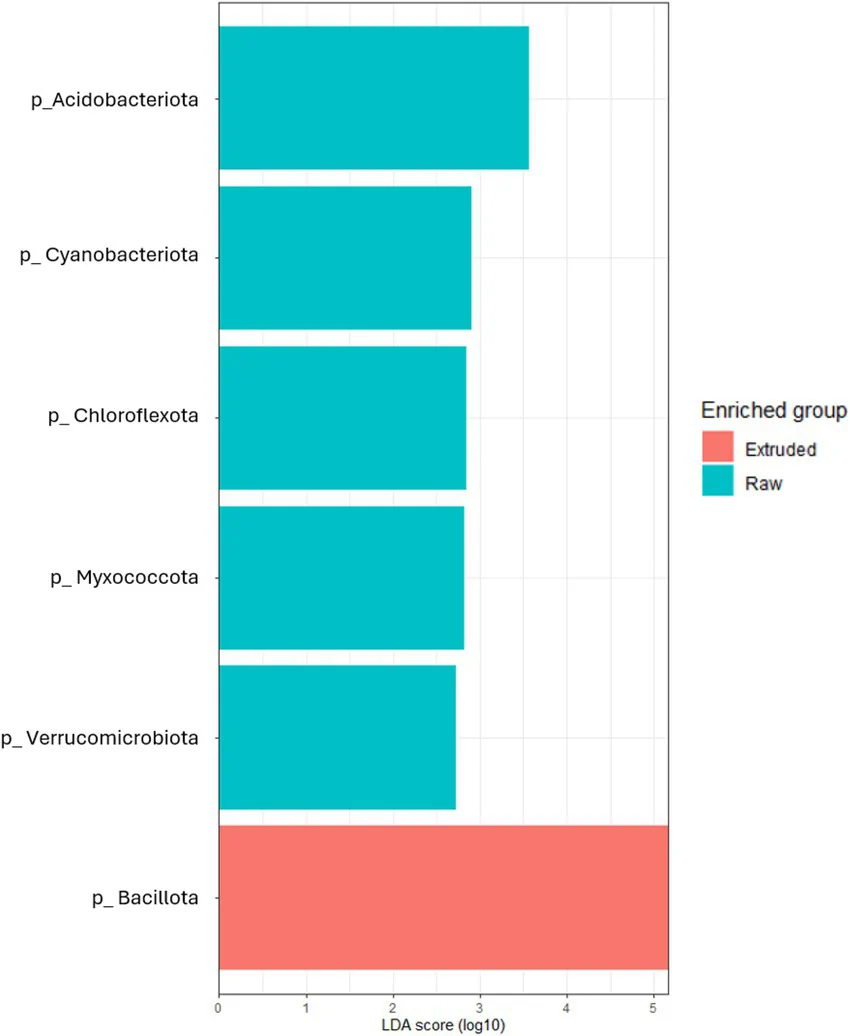
LEfSe effect-size profile of bacterial phyla nominally discriminative between extruded-fed and raw-fed dogs under the original LEfSe criteria (Kruskal–Wallis *p* < 0.05, Wilcoxon *p* < 0.05, and LDA score ≥ 2). Phylum-level signals did not remain significant after Benjamini–Hochberg correction across all 35 phylum-level tests (minimum *q* = 0.066).

Across all samples, the gut microbiota was dominated by the classes Clostridia, Fusobacteriia, and Bacteroidia (Supplementary Figure S4), and at the order level, Fusobacteriales, Bacteroidales, and Lachnospirales were predominant (Supplementary Figure S5). Fusobacteriaceae, Bacteroidaceae, and Lachnospiraceae were the most abundant families (Supplementary Figure S6). Under the original nominal LEfSe criteria, four families were nominally discriminative for extruded-fed dogs and 25 for raw-fed dogs (Figure 6A and Supplementary Table S1). At the genus level, the microbiota was dominated by *Fusobacterium*, followed by *Bacteroides* and *Prevotella*, with additional contributions from *Clostridium* sensu stricto, *Peptostreptococcus*, and other less abundant taxa. LEfSe identified six genera as nominally discriminative for extruded-fed dogs, including *Prevotella* and *Catenibacterium*, and 29 genera as nominally discriminative for raw-fed dogs, including *Paeniclostridium* (Figure 6B and Supplementary Table S1). However, none of the family- or genus-level signals remained significant after Benjamini–Hochberg correction across all 240 family-level and 477 genus-level tests (minimum *q* = 0.070 and 0.150, respectively). Accordingly, these taxon-level findings are considered exploratory nominal signals rather than FDR-supported differential taxa.

**Figure 6:**
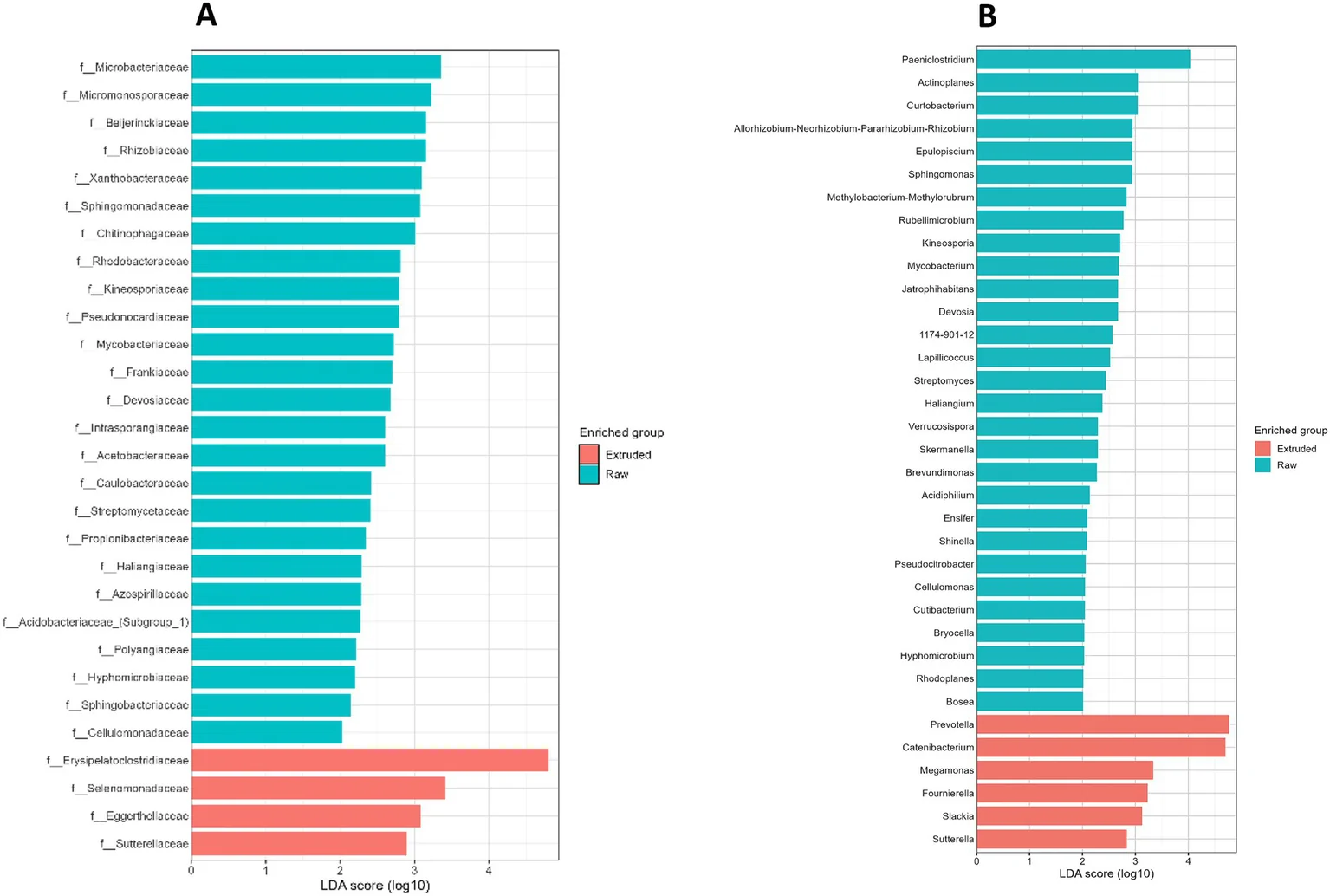
LEfSe effect-size profiles of bacterial families **(A)** and genera **(B)** nominally discriminative between extruded-fed and raw-fed dogs under the original LEfSe criteria (Kruskal–Wallis *p* < 0.05, Wilcoxon *p* < 0.05, and LDA score ≥ 2). None of these family- or genus-level signals remained significant after Benjamini–Hochberg correction across all 240 family-level and 477 genus-level tests (minimum *q* = 0.070 and 0.150, respectively).

Additionally, a targeted LEfSe analysis was conducted focusing on *Clostridium* related genera due to their relevance in animal and public health. *Clostridium* sensu stricto 1 and *Clostridium* sensu stricto 12 showed higher relative abundances in raw-fed dogs; however, these differences did not reach statistical significance. *Clostridium* sensu stricto 1 exhibited a higher relative abundance in raw-fed dogs compared with extruded-fed dogs (Supplementary Figure S7).

### 3.7 Association between fecal pathogen carriage and gut microbiota α and β diversity in raw-fed dogs

Statistically significant differences were observed in the Observed Species, Chao1, ACE and Fisher diversity indices between raw-fed pets classified as pathogen carriers and non-pathogen carriers (*n* = 4) (Figure 7). For the beta diversity analysis, no significant differences were found between groups (*p* = 0.141; PERMANOVA analysis) (Supplementary Figure S8). However, results should be interpreted cautiously due to the limited sample size.

**Figure 7:**
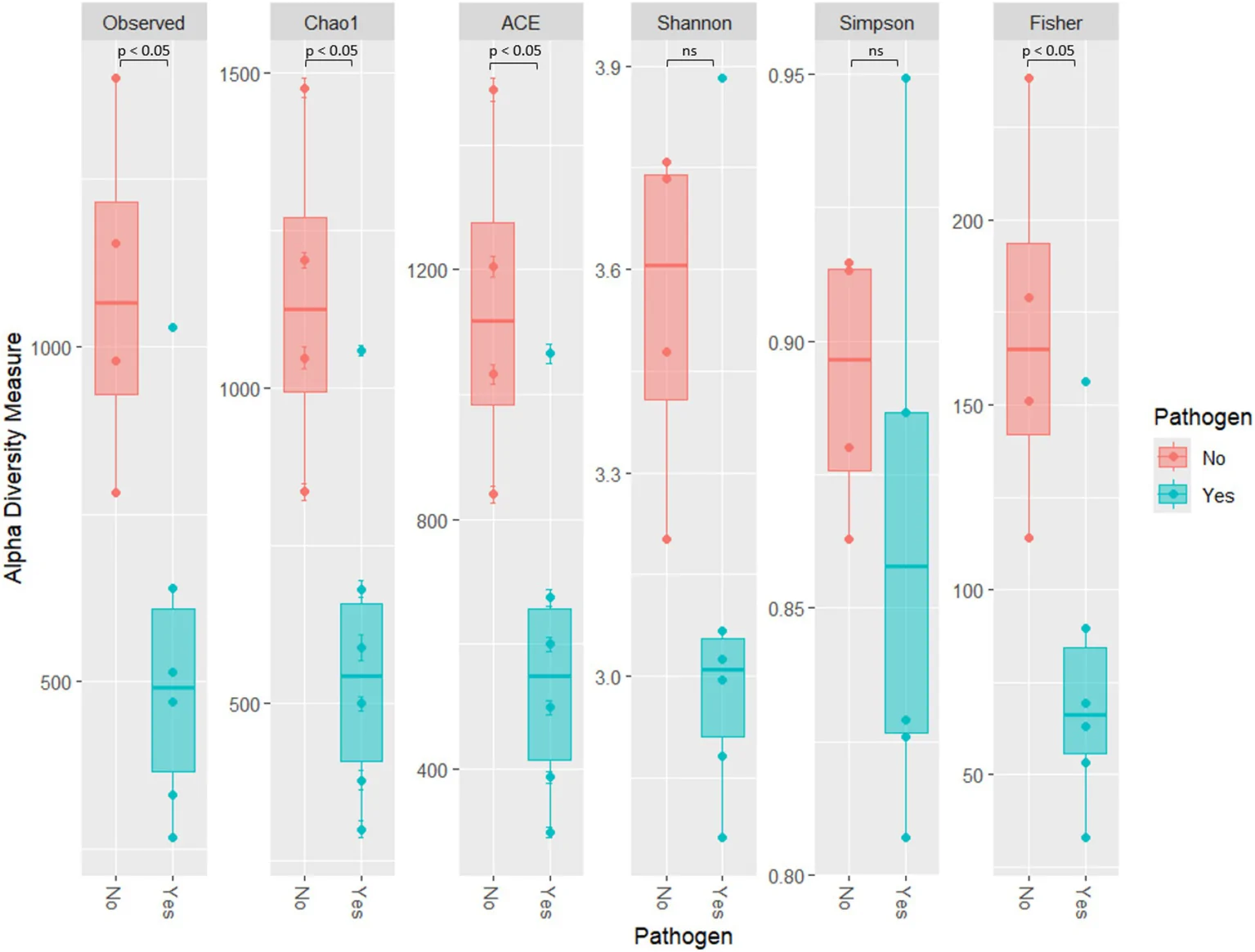
Comparison of alpha diversity indices (Observed species, Chao1, ACE, Shannon, Simpson, and Fisher) between raw-fed dogs classified as pathogen carriers (*Salmonella* carriers = 5; *L. monocytogenes* carriers = 1) and non-pathogen carriers (*n* = 4). Statistical differences between groups were assessed using the Mann–Whitney *U* test.

## 4 Discussion

Given the growing interest of pet owners in feeding their pets unconventional diets such as raw diets (Dodd et al., 2020; Solís et al., 2022) it is essential to study how each type of diet according to its properties, such as format and nutritional composition, shapes the gastrointestinal microbiota of pets and how these changes might relate to health or disease. In this study we first analyzed the nutritional composition of extruded and raw diets. Subsequently, we compared the gut microbiota composition of dogs fed extruded and raw diets and further explored whether fecal carriage of zoonotic pathogens (i.e., *Salmonella* and *L. monocytogenes*) was associated with modifications in the fecal microbial composition in dogs fed raw diets. From a One Health perspective, understanding the way diet influences the canine gut microbiota is particularly important since pets share close physical environments with humans. This close contact facilitates microbial exchange between pets and humans.

Regarding diet composition, laboratory analyses were conducted to verify the accuracy of the declared nutritional content of the diets and to ensure a reliable characterization of their composition. Results revealed distinct macronutrient profiles among diets, in addition to discrepancies between the information listed on each product’s label. These findings highlight the greater compositional heterogeneity of raw diets compared to extruded products and the potential impact these imbalances could have on pets’ health (Vecchiato et al., 2022). Different studies have reported nutritional imbalances in pet food, particularly in raw food, however, these studies have not compared the deviations identified with the nutrients declared on the product label (Vecchiato et al., 2022; Lyu et al., 2025; Pedrinelli et al., 2017).

While organizations such as the Association of American Feed Control Officials (AAFCO) and the European Pet Food Industry Federation (FEDIAF) provide recommendations for the nutritional requirements and quality standards of dog and cat foods, their guidelines do not specify acceptable analytical tolerances for discrepancies between nutrient concentrations declared on product labels and those determined by laboratory analysis (European Pet Food Industry Federation [FEDIAF], 2025; Association of American Feed Control Officials [AAFCO], 2025).

In Chile, pet foods are regulated under the Animal Feed Regulation (Decree No. 4/2016) and must comply with the Chilean Standard NCh2546.Of2001 regarding composition and labeling. However, these regulations do not establish acceptable analytical tolerances between declared label values and laboratory results. Therefore, the technical tolerances provided in Regulation (EC) No. 767/2009 are frequently used as a reference for interpreting analytical compliance, particularly in countries where such criteria are not defined in national legislation (Ministerio de Agricultura, 2016; European Parliament C of the EU, 2018). The identified inconsistencies reflect the necessity of enhancing quality-control measures in pet food manufacturing in order to improve the reliability of nutrient formulation, and the labeling information.

Beyond the identified discrepancies in labeling accuracy, given the differences in nutrient composition between diets, we subsequently examined whether the dietary groups were associated with distinct fecal microbial profiles. In this regard, the analyzed diets also showed different macronutrient profiles. RMBDs contained higher concentrations of crude protein and fat content and lower concentrations of fiber and non-fiber carbohydrates than extruded diets. These differences are microbiologically relevant since the amount and type of substrates reaching the gastrointestinal tract can favor the predominance of specific microorganisms (Campbell et al., 2026). While diets high in carbohydrates and fiber may promote saccharolytic fermentation and the synthesis of short-chain fatty acids, diets high in protein may provide more nitrogen-containing substrates for proteolytic fermentation (Badri et al., 2021).

In terms of overall bacterial taxa, our results are consistent with previous reports describing Bacillota, Bacteroidota, and Fusobacteriota as the predominant bacterial phyla in the canine gut, regardless of dietary type (Kim et al., 2025; Garcia-Mazcorro and Minamoto, 2013; Minamoto et al., 2019; Moon et al., 2018). However, their relative abundances differed between dietary groups in the present study, with raw-fed dogs showing a higher abundance of Fusobacteriota and extruded-fed dogs showing a higher abundance of Bacillota. This pattern is expected given that Fusobacteriota has a greater capacity to degrade proteins into amino acids and peptides, while Bacillota are associated with the breakdown of complex carbohydrates (Flint et al., 2012; Garrigues et al., 2022; Kim et al., 2025). Although, the observed relative-abundance patterns and nominal LEfSe signals are associated with dietary groups, they cannot distinguish between the effects of macronutrient profile and/or food format on microbiota composition. Because the taxon-level LEfSe signals did not remain significant after FDR correction, they should be interpreted as exploratory rather than confirmatory evidence of differential taxa.

Moreover, raw-fed dogs showed a higher relative abundance of phyla described as low-abundance microorganisms (<1%) compared to extruded-fed dogs. For instance, Verrucomicrobiota, a low-abundance bacterial phylum, has been identified in fecal samples from stray dogs, usually characterized by a highly varied diet (Li et al., 2022; Zhou et al., 2024).

At the genus level, *Prevotella* and *Catenibacterium* were among the nominally discriminative LEfSe signals associated with extruded-fed dogs. *Prevotella* is related to the degradation of fiber and carbohydrates, whereas *Catenibacterium* comprises strictly anaerobic, sugar-fermenting bacteria that produce short-chain fatty acids as products of glucose metabolism (Campbell et al., 2026; Gerges et al., 2024; Pilla and Suchodolski, 2021). Lower relative abundance of *Catenibacterium* in raw-fed dogs has previously been described in the context of shifts from carbohydrate-rich to protein-rich diets (Xu et al., 2021).

Additionally, *Paeniclostridium* was likewise identified as a nominally discriminative genus associated with raw-fed dogs. This genus was previously classified among *Clostridium* members and includes species associated with gas gangrene, enteric or histotoxic infections, and hemorrhagic and necrotizing gastroenteropathy in animals (Nyaoke et al., 2020; Gonzalez-Astudillo et al., 2023; Capewell et al., 2020). Although its presence does not directly imply disease (Vidor et al., 2019), its identification as a nominally discriminative genus in raw-fed dogs may warrant attention, given that members of this genus have been associated with disease in several animal species. However, because no genus-level association remained significant after Benjamini-Hochberg correction, the *Paeniclostridium* signal should be considered exploratory and should not be interpreted as evidence of increased pathogen-related risk. Even though, taxon-level LEfSe findings should be considered exploratory, these results should be interpreted separately from the weighted UniFrac PERMANOVA, which evaluates community-level compositional differences.

Among the identified *Clostridium sensu stricto* clusters, cluster 1 was observed at higher relative abundance in dogs fed raw diets than those fed extruded diets. The bacterial class Clostridia and order Clostridiales are very diverse at the genera and species level and might include beneficial as well as pathogenic bacteria (Suchodolski, 2021). For example, *Clostridium sensu stricto* 1 might comprise both human and animal pathogens, such as *Clostridium perfringens, Clostridium tetani, Clostridium botulinum* and other real *Clostridium* species (Yang et al., 2019; Gupta and Gao, 2009). In humans, whose gut microbiome is more similar to that of dogs than to that of other animals such as pigs or mice (Coelho et al., 2018) members of *Clostridium sensu stricto* 1 are often considered pathogenic and indicators of gut dysbiosis (Wang et al., 2019).

The genus *Helicobacter* was identified in both dietary groups as part of core microbiota. Although members of this genus include microorganisms with pathogenic and zoonotic potential, non-*Helicobacter pylori* species are common colonizers of the canine gastrointestinal tract. Therefore, the identification of this genus from canine fecal samples should not be interpreted as evidence of gastrointestinal disease. However, some canine, and also feline, *Helicobacter* species have been detected in humans and have been associated with gastric disease (Taillieu et al., 2022).

Although the taxonomic resolution provided by V3-V4 16S rRNA gene sequencing does not allow reliable species or strain identification or the identification of its virulence, the identification of bacterial genera that can cause disease in humans emphasizes the significance of their impact from a One Health perspective (Taillieu et al., 2022; Johnson et al., 2019).

The higher estimate of species richness identified in the raw-fed group compared to extruded-fed group might be explained by the differences in the availability of fermentable substrates and nutrient sources which influence community structure and bacterial metabolism (Li et al., 2017). Wu et al. (2022) for instance, showed that higher proportion of dietary protein provides substrates for distinct bacterial groups, contributing to increased species diversity and abundance. Regarding beta diversity, analyses revealed differences in microbiota composition between groups, indicating that these differences are not solely due to the presence of specific species but also involve variations in their relative abundances (Chang et al., 2011).

Furthermore, fecal samples from canine pathogen carriers previously identified as raw-fed dogs (Solís et al., 2022) were included in this study to assess the association between pathogen carriage and microbiota composition. The findings suggest that, beyond the diet-mediated modulation of the microbiota, the presence of pathogens might have an important influence on the gut microbiota of pets, even when fed the same diet. Although association between fecal pathogen carriage and alpha diversity was observed, no significant differences in beta diversity were detected suggesting that pathogen carriage may be associated with changes in microbial diversity rather than in the overall community structure (Yin et al., 2026).

These changes in microbial diversity may be partly explained by alterations in colonization resistance, a key microbiota-mediated defense mechanism that limits pathogen establishment through nutrient and niche competition, production of antimicrobial compounds, generation of microbial-derived metabolites, and modulation of host immune responses. Enteric pathogens, such as *Salmonella*, might overcome colonization resistance by reshaping the intestinal environment, promoting inflammation, using nutrients and niches, among other mechanisms. Also, diet can induce changes in microbial communities and their metabolites and may either strengthen resistance against pathogen colonization or compromise these protective mechanisms, thereby increasing susceptibility to enteric pathogens (Deng and Wang, 2024).

In this regard, raw diets may act as modulators of colonization resistance because they induce marked changes in the composition and metabolic activity of the canine gut microbiota. These changes might alter microbial interactions and ecological niches, which might contribute to resistance against enteric pathogen colonization (Castañeda et al., 2023). To our knowledge, while the influence of pathogens on the gut microbiota has been observed in humans, this study represents one of the first investigations into the relationship between fecal pathogen carriage and fecal microbiota richness in dogs. These results highlight the importance of integrating the One Health concept into veterinary decision-making by integrating public health considerations into nutritional recommendations. Applying a One Health approach may contribute to reducing the risk of pathogen shedding into the environment, thereby minimizing potential transmission between pets, humans, and the environment (Pegg et al., 2024).

From a practical veterinary nutrition point of view, these findings support the importance of guiding nutritional recommendations based on a comprehensive assessment comprising diet composition, labeling reliability, individual patient needs, and food safety. RMBDs may require greater attention to microbiological risks and nutritional consistency, especially in households that include children, older adults, immunocompromised individuals, and even other susceptible animals (Bernaquez et al., 2025).

Regarding the risk of gastrointestinal disease in pets, while a healthy gut microbiota acts as a defense mechanism against zoonotic microorganisms, certain pathogens, such as *Salmonella*, can overcome these gastrointestinal defenses (Rogers et al., 2024). However, pathogen colonization does not entirely depend on the exposure through diet; modifications in the intestinal environment should be considered since they may create favorable conditions for pathogen persistence within the gut. Badri et al. (2021), for example, described that consuming high-protein diets, such as raw diets, might produce toxic metabolites and increase proteolytic fermentation, which can lead to potential damage to the intestinal mucosa and create an environment more conducive to pathogen colonization.

An increased microbial diversity is often associated with better health outcomes (Huang et al., 2020), however, it is essential to recognize that the relationship between microbial diversity and health is not linear and depends on the ecological and functional context of the microbiome (Williams et al., 2024). In the present study, although raw-fed dogs exhibited higher overall microbial richness and diversity compared to extruded-fed dogs, those identified as fecal pathogen carriers within the raw-fed group showed significantly reduced alpha diversity indices. These findings suggest that increased diversity does not necessarily reflect a uniformly beneficial microbial profile and may mask the presence of potentially pathogenic taxa.

The greater abundance and diversity of taxa should also be linked to the type of diet consumed since raw foods, that have not undergone heat treatment, already have a higher microbial load and an increased risk of pathogenic bacterial contamination in comparison with extruded diets (Solís et al., 2022; Soffer et al., 2016). Kim et al. (2017) for instance reported that the increased diversity in raw-fed dogs could also elevate the risk of opportunistic infections due to higher levels of certain bacteria, since dogs fed a raw diet are more likely to be exposed to pathogens such as *Salmonella,* pathogenic *E. coli,* and *Clostridium perfringens*, among others (Solís et al., 2022).

The authors acknowledge that dietary groups were not completely balanced by age, with three puppies included in the extruded-fed group. However, a sensitivity analysis excluding these animals maintained the overall alpha and beta diversity patterns, suggesting that their inclusion was not the primary driver of the observed differences. Nevertheless, residual confounding by other host-related factors cannot be excluded. Although the number of diets was relatively small and unequal between groups (6 RMBDs vs. 10 extruded diets), which may limit the generalizability of the nutritional comparisons, the observed multivariate differences remained highly significant and stable in the leave-one-RMBD-out sensitivity analysis, suggesting that the findings were not driven by any single RMBD.

In addition, the pathogen-carriage analysis included only six carriers and four non-carriers, limiting statistical power, particularly for beta diversity analyses, therefore the non-significant beta diversity result should not be interpreted as evidence of similarity between carriers and non-carriers. Given the cross-sectional design, the direction of the association between pathogen carriage and reduced microbial richness should be considered exploratory and also as a basis for future research. Regardless of the above, the present results have important implications for both the potential transmission of pathogens to humans (Davies et al., 2019; Finley et al., 2007) and the risk of gastrointestinal disease in pets (Boneva-Marutsova et al., 2025).

Lastly, beyond simply identifying increases or decreases in specific taxa, future studies should focus on interpreting microbial interactions, production of metabolites, and the association of the findings with pet health. Also, larger samples and longitudinal sampling to determine the temporal correlations between diet, microbiota, and pathogen carriage would be valuable. Importantly, such research should aim to translate these findings into practical and accessible diagnostic tools that can be easily implemented in clinical settings, therefore improving the assessment of gut health in companion animals.

## 5 Conclusion

The present study identified nutritional differences between the raw meat-based diets and extruded diets, while discrepancies between declared and analyzed nutrient composition were more frequently observed among the RMBDs evaluated. These findings highlight that the nutrient composition declared on pet food labels may not always reflect the real composition of the product. This emphasizes the need for better quality-control procedures and greater consistency between formulation, manufacturing, and labeling within the pet food industry. From a practical perspective, veterinarians and pet owners should therefore consider not only the nutrient information provided on the label but also the reliability, transparency, and quality-control practices of pet food manufacturers when selecting commercial diets. Diet was also associated with differences in canine fecal microbiota composition, although alpha diversity patterns varied depending on the metric evaluated. Specific nominally discriminative taxa were identified for extruded and raw-fed dogs which strengthen previously published studies on the effect of diet on the composition of the microbiota. The detection of potentially zoonotic microorganisms further emphasizes the relevance of these considerations within a One Health context. Importantly, our results emphasize the potential role of pathogens, such as *Salmonella* and *L. monocytogenes*, in shaping microbiota diversity as well as the influence of diet on the gut environment in facilitating pathogen establishment, highlighting the interplay between diet, microbiota, and host health. These findings revealed the need for further research on the relationship between pathogen carriage and microbiota composition, its potential impact on pet health and public health. Although increased microbial diversity in raw-fed dogs was noted, it is important to recognize that the relationship between microbial diversity and health is complex, as greater diversity does not always equate to a balanced or healthy gut microbiota.

## Data Availability

The raw data produced from DNA sequencing in this study were deposited in the ENA-EMBL database under the accession number PRJEB111146 (https://www.ebi.ac.uk/ena/browser/view/PRJEB111146).

## Supplementary material

SUPPLEMENTARY FIGURE S1 Principal component analysis (PCA) of nutrient composition in raw meat-based diets (RMBDs; *n* = 6) and extruded diets (*n* = 10). PC1 and PC2 explained 68.09% and 25.09% of the total variance, respectively (93.18% cumulative variance). Ellipses represent 95% confidence regions for each diet group (MANOVA: Pillai's trace = 0.977, *F*(4,11) = 118.86, *p* < 0.001).

SUPPLEMENTARY FIGURE S2 Non-rarefied curves of amplicon sequence variants (ASVs) diversity in samples from raw-fed dogs (RD; blue) and extruded-fed dogs (ED; red).

SUPPLEMENTARY FIGURE S3 Sensitivity analysis of beta diversity after exclusion of puppies. Principal coordinate analysis (PCoA) based on weighted UniFrac distances comparing the fecal microbiota of adult and senior dogs fed extruded (*n* = 11) or raw diets (*n* = 10), after exclusion of the three puppies from the extruded-fed group. Microbial community composition remained significantly associated with diet type (PERMANOVA: *R*² = 0.168, *F* = 3.838, *p* = 0.006), with no evidence of differences in multivariate dispersion between groups (PERMDISP: *F* = 0.213, *p* = 0.635).

SUPPLEMENTARY FIGURE S4 Overview of bacterial classes identified in the extruded and raw-fed groups.

SUPPLEMENTARY FIGURE S5 Overview of bacterial orders identified in the extruded and raw-fed groups.

SUPPLEMENTARY FIGURE S6 Overview of bacterial families identified in the extruded and raw-fed groups.

SUPPLEMENTARY FIGURE S7 Relative abundance of *Clostridium* sensu stricto 1 in dogs fed extruded and raw diets. No significant difference was detected between dietary groups.

SUPPLEMENTARY FIGURE S8 PCoA plot of beta diversity values based on weighted UniFrac distances. Comparison of the fecal microbiota between raw-fed pathogen carriers (yes: blue dots) and non-carrier dogs (no: red dots). Dots represent the samples, and different colors represent each group. The closer the distance between samples, the higher the similarity. Ellipses show confidence intervals (CI) of 95% for each sample type (*p* = 0.141; PERMANOVA analysis).
